# Short course daily prednisolone therapy during an upper respiratory tract infection in children with relapsing steroid-sensitive nephrotic syndrome (PREDNOS 2): protocol for a randomised controlled trial

**DOI:** 10.1186/1745-6215-15-147

**Published:** 2014-04-27

**Authors:** Nicholas J A Webb, Emma Frew, Elizabeth A Brettell, David V Milford, Detlef Bockenhauer, Moin A Saleem, Martin Christian, Angela S Hall, Ania Koziell, Heather Maxwell, Shivram Hegde, Eric R Finlay, Rodney D Gilbert, Jenny Booth, Caroline Jones, Karl McKeever, Wendy Cook, Natalie J Ives

**Affiliations:** 1 Department of Paediatric Nephrology and NIHR/Wellcome Trust Children's Clinical Research Facility, University of Manchester, Manchester Academic Health Science Centre, Royal Manchester Children's Hospital, Manchester, UK; 2 Health Economics Unit, University of Birmingham, Birmingham, UK; 3 Birmingham Clinical Trials Unit, University of Birmingham, Birmingham, UK; 4 Department of Paediatric Nephrology, Birmingham Children’s Hospital, Birmingham, UK; 5 Department of Paediatric Nephrology, Great Ormond Street Hospital, London, UK; 6 Children’s and Academic Renal Unit, University of Bristol, Bristol, UK; 7 Department of Paediatric Nephrology, Nottingham Children’s Hospital, Nottingham, UK; 8 Children’s Hospital, University Hospitals of Leicester, Leicester, UK; 9 Department of Paediatric Nephrology, Evelina Children’s Hospital, London, UK; 10 Renal Unit, Royal Hospital for Sick Children, Glasgow, UK; 11 Children's Kidney Centre, University Hospital of Wales, Cardiff, UK; 12 Department of Paediatric Nephrology, Leeds Children’s Hospital, Leeds, UK; 13 Department of Paediatric Nephrology, University Hospital Southampton, Southampton, UK; 14 Department of Paediatric Nephrology, Great North Children’s Hospital, Newcastle upon Tyne, UK; 15 Department of Paediatric Nephrology, Alder Hey Children’s Hospital, Liverpool, UK; 16 Department of Paediatric Nephrology, Royal Belfast Hospital for Sick Children, Belfast, UK; 17 Nephrotic Syndrome Trust, Bristol, UK; 18 National Institute for Health Research Medicines for Children Research Network Nephrology Clinical Studies Group, UK

**Keywords:** Steroid sensitive nephrotic syndrome, Relapse, Upper respiratory tract infection, Prednisolone, Adverse effects, Health economic analysis

## Abstract

**Background:**

Relapses of childhood steroid-sensitive nephrotic syndrome (SSNS) are treated with a 4- to 8-week course of high-dose oral prednisolone, which may be associated with significant adverse effects. There is a clear association between upper respiratory tract infection (URTI) and relapse development. Previous studies in developing nations have suggested that introducing a 5- to 7-day course of daily prednisolone during an URTI may prevent a relapse developing and the need for a treatment course of high-dose prednisolone. The aim of PREDNOS 2 is to evaluate the effectiveness of a 6-day course of daily prednisolone therapy during an URTI in reducing the development of a subsequent relapse in a developed nation.

**Methods/design:**

The subjects will be 300 children with relapsing SSNS (≥2 relapses in preceding year), who will be randomised to receive either a 6-day course of daily prednisolone or no change to their current therapy (with the use of placebo to double blind) each time they develop an URTI over 12 months. A strict definition for URTI will be used. Subjects will be reviewed at 3, 6, 9 and 12 months to capture data regarding relapse history, ongoing therapy and adverse effect profile, including behavioural problems and quality of life. A formal health economic analysis will also be performed. The primary end point of the study will be the incidence of URTI-related relapse (3 days of Albustix +++) following the first infection during the 12-month follow-up period. DNA and RNA samples will be collected to identify a potential genetic cause for the disease. Subjects will be recruited from over 100 UK centres with the assistance of the Medicines for Children Research Network.

PREDNOS 2 is funded by the National Institute for Health Research Health Technology Assessment Programme (11/129/261).

**Discussion:**

We propose that PREDNOS 2 will be a pivotal study that will inform the future standard of care for children with SSNS. If it is possible to reduce the disease relapse rate effectively and safely, this will reduce the morbidity and cost associated with drug treatment, notwithstanding hospital admission and parental absence from employment.

**Trial registration:**

Current Controlled Trials (ISRCTN10900733).

## Background

Idiopathic nephrotic syndrome is the commonest glomerular disease of childhood, with an incidence of 2 cases per 100,000 children in the UK [1]. The presenting episode is treated with high-dose oral prednisolone to which >90% make a complete response, responders receiving the diagnostic label of steroid-sensitive nephrotic syndrome (SSNS) [2,3]. The optimum duration of prednisolone therapy at presentation remains unclear and is currently being further investigated in the PREDNOS study (ISRCTN16645249).

Following successful initial treatment, 70% to 80% of children develop disease relapses necessitating further 4- to 8-week courses of high-dose prednisolone, and around 50% develop frequently relapsing disease [4]. Various long-term immunosuppressive strategies are employed to reduce the frequency of relapses in those with frequently relapsing disease. These include long-term low-dose alternate-day prednisolone, levamisole, cyclophosphamide, ciclosporin, tacrolimus, mycophenolate mofetil and rituximab.

Nephrotic syndrome relapses are associated with a risk of significant complications, including sepsis, thrombosis, dyslipidaemia and malnutrition [5]. The treatment of relapses with high-dose prednisolone is associated with major adverse effects, including hip avascular necrosis, hypertension, diabetes and behavioural problems [6]. Furthermore, children miss school during relapses, resulting in impaired academic performance and parental absence from work. It is well recognised that at least 50% of relapses are precipitated by a viral upper respiratory tract infection (URTI), possibly mediated through cytokine release [7]. Given this causal association, and the morbidity and cost associated with relapse and its treatment, it is logical that attempts are made to ameliorate the URTI-driven process.

Three studies published to date suggest that in children from Middle Eastern and Asian countries (Saudi Arabia, Sri Lanka and India) with relapsing SSNS receiving long-term alternate-day prednisolone therapy, the use of a 5- to 7-day course of daily prednisolone therapy (i.e. switching from alternate day to daily dosing) at the time of development of intercurrent URTI associated with fever is associated with a lower rate of subsequent relapse of nephrotic syndrome than when no such change is made to their therapy (current standard care) [8-10]. There were, however, a number of methodological issues with some of these studies, and the strictly defined patient populations under investigation limit the applicability of the study findings to children with SSNS in developed nations, where the pattern of childhood URTI is significantly different, with lower incidence of fever and general absence of diarrhoea. Furthermore, these previous studies have been restricted to children receiving low- to medium-dose long-term maintenance prednisolone (plus levamisole in the Indian study [10]) and it is currently not clear whether intervention with a short course of daily prednisolone at the time of URTI is effective in children receiving other immunomodulatory therapies, e.g. levamisole, ciclosporin, tacrolimus and mycophenolate mofetil, either alone, or in conjunction with long-term maintenance prednisolone. The adverse effects of prednisolone, including its effect on behaviour, were not comprehensively assessed, nor were quality of life or the cost-effectiveness of the intervention. The PREDNOS 2 study will provide important definitive evidence to assess the effectiveness of a 6-day course of daily prednisolone therapy at the time of development of URTI in reducing the development of subsequent nephrotic syndrome relapse in children with relapsing SSNS in a developed nation.

## Methods/design

**Figure 1:**
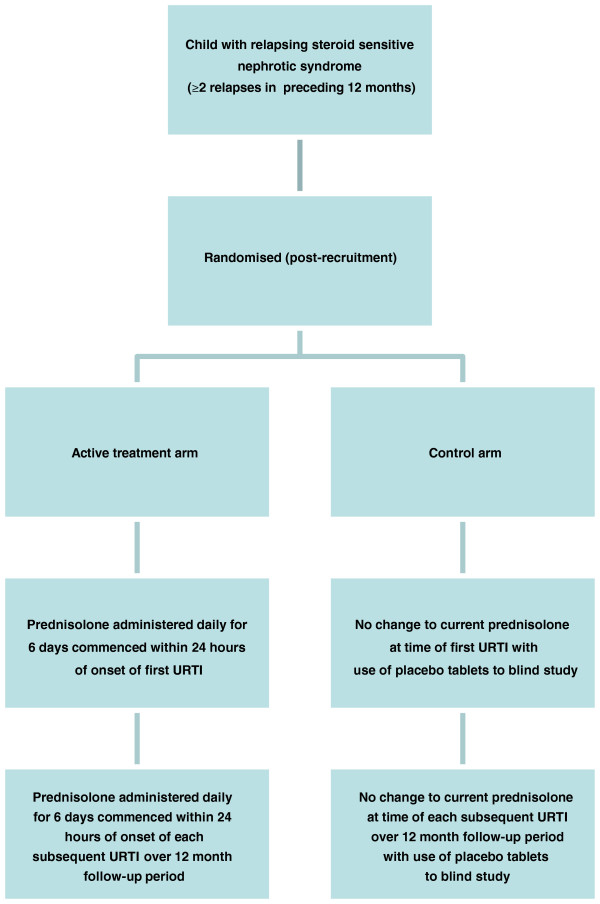
Trial schema.

A flow chart for the study is shown in Figure 1.

### Aim

The aim of the study is to assess the effectiveness of a 6-day course of daily prednisolone therapy at the time of onset of URTI in reducing the development of subsequent nephrotic syndrome relapse in children with relapsing SSNS.

### Study design

PREDNOS 2 will be conducted as a phase III randomised parallel-group placebo-controlled double-blind trial, comparing a 6-day course of daily prednisolone with no change in therapy using a matching placebo. The study duration will be 4 years, comprising 6 months for study set-up, 2 years of recruitment, 1 year of follow-up and 6 months for data analysis.

### Recruitment

Children with relapsing SSNS under the care of a paediatric nephrologist and/or a general paediatrician will be recruited from over 100 centres throughout the UK. Potential subjects will be identified from clinic lists and departmental databases. Information sheets outlining the study will be mailed to the parents or guardians of potentially eligible children (and the child where age appropriate), 1 to 2 weeks prior to their next clinical appointment. Following confirmation of eligibility with regard to inclusion and exclusion criteria (see below) and a further full discussion of the study, informed consent will then be sought from the parents (or guardians) and subjects (informed consent or assent according to age) at the time of this review.

### Inclusion and exclusion criteria

Inclusion criteria:

• Diagnosis of relapsing SSNS with two or more relapses in the 12 months prior to enrolment

• Age 2 to 19 years inclusive

• Parents/guardians and subject (where age appropriate) understand the definition of URTI and the need to commence the study drug once this definition has been met

• Written informed consent obtained from the subject’s parents/guardians and written assent obtained from subject (where age appropriate). Subjects aged 16 years and above will provide their own written informed consent

• Subjects who have previously received a course of oral or intravenous cyclophosphamide:

○ Must have experienced two relapses in the 12 months prior to randomisation (in keeping with all other subjects)

○ Must have experienced at least one of these relapses following completion of cyclophosphamide therapy

○ Must be at least 3 months post completion of oral or intravenous cyclophosphamide therapy

• Subjects who have previously received a single dose or course of intravenous rituximab:

○ Must have experienced two relapses in the 12 months prior to randomisation (in keeping with all other subjects)

○ Must have experienced at least one of these relapses following completion of rituximab therapy

○ Must be at least 3 months post completion of intravenous rituximab therapy

Subjects with a diagnosis of relapsing SSNS will include:

• Subjects not on long-term immunomodulatory therapy

• Subjects receiving long-term maintenance prednisolone therapy at a dose of up to and including 15 mg/m^2^ on alternate days

• Subjects receiving long-term maintenance prednisolone therapy at a dose of up to and including 15 mg/m^2^ on alternate days in conjunction with other immunomodulatory therapies, including levamisole, ciclosporin, tacrolimus, mycophenolate mofetil, mycophenolate sodium and azathioprine

• Subjects receiving long-term immunomodulatory therapies, including levamisole, ciclosporin, tacrolimus, mycophenolate mofetil, mycophenolate sodium and azathioprine without long-term maintenance prednisolone therapy

Exclusion criteria:

• Steroid-resistant nephrotic syndrome

• Receiving or within 3 months of completion of a course of oral or intravenous cyclophosphamide

• Receiving or within 3 months of completion of a course of rituximab

• Daily prednisolone therapy

• Subjects on a prednisolone dose of greater than 15 mg/m^2^ on alternate days

• Documented history of significant non-adherence with medical therapy

• Planned transfer from paediatric to adult services during the 12-month study period

• Inability to take prednisolone tablets, even in crushed form

• Known allergy to prednisolone

Daily prednisolone therapy or alternate-day prednisolone therapy refers to a dose of prednisolone at the time of randomisation only. If the prednisolone dose increases during the 12-month study period, e.g. after a relapse, the subject may remain in the study.

### Randomisation and allocation concealment

Following the completion of the baseline assessments, subjects will be randomised at the level of the individual on a 1:1 basis to either the active treatment or control arm. Randomisation will be provided by a computer-generated programme at the University of Birmingham Clinical Trials Unit (BCTU). The randomisation will be stratified by the treatment regimen that the subject is receiving at randomisation (no treatment, long-term maintenance prednisolone therapy only, long-term maintenance prednisolone therapy with other immunosuppressant therapy and other immunosuppressant therapy only). Treatment allocation will only be revealed to the central pharmacy dispensing the study drug at Birmingham Children’s Hospital.

The study drug (prednisolone 5 mg tablets and matching placebo) will be manufactured, packed and labelled by Essential Nutrition. Following randomisation, subjects will be provided with a supply of 100 tablets (two containers of 50 tablets) of the study drug (prednisolone 5 mg for those randomised to the active treatment arm or a matching placebo for those randomised to the control arm). This will be sent directly to the family home from the central pharmacy at the Birmingham Children’s Hospital by Royal Mail registered post on a day convenient for the family. The study drug containers and their contents will appear identical in every way, thus maintaining the double blind. Study drug labelling will comply with the applicable regulatory requirements and clinical trial specific labels will be attached to all treatments. Birmingham Children’s Hospital Pharmacy will maintain drug accountability logs for dispensed and returned study drug doses according to their local policy.

### Planned interventions

Current clinical practice is for no change to be made to immunomodulatory therapy at the time of development of an URTI. The intervention being assessed within PREDNOS 2 is a 6-day course of daily prednisolone therapy at the time of onset of URTI. Those randomised to the *active treatment arm* will commence a 6-day course of daily prednisolone each time they develop an URTI (see below for definition) during the 12 months of the trial. Those randomised to the *control arm* will receive an identical number of placebo tablets, thus allowing double blinding of the study. If the subject is receiving background long-term immunomodulatory therapy (e.g. levamisole or ciclosporin), then this will continue unchanged (see below for examples).

### Definition of an upper respiratory tract infection

An URTI will be defined by the presence of at least two of the following *for at least 24 hours*:

• Sore throat

• Ear pain or discharge

• Runny nose

• Cough (dry or barking)

• Hoarse voice

• Fever >37°C (measured using a tympanometric electronic thermometer)

Parents or guardians will be provided with clear written and (if requested) downloadable electronic information regarding the study definition of an URTI as outlined above. They will also be provided with an abbreviated version printed onto laminated cards and in a fridge magnet format. An electronic tympanometric thermometer will be provided to facilitate measurement of their child’s temperature, as well as a study diary to record the results of the daily morning urinalysis (standard care in children with relapsing SSNS to allow the early identification of disease relapse), development of URTI, commencement of study drug, other ongoing treatment, acute illnesses and any other issues they feel might be important for the trial.

### Commencement of study drug

Once the subject meets the definition for URTI (two or more criteria as listed above for at least 24 hours), the parents or guardians will commence the subject on the study drug (prednisolone tablets for those in the active treatment arm or placebo for those in the control arm; see below for dosing schedules). It is anticipated that parents or guardians will have no difficulties in identifying that their child has met the URTI criteria and will commence the study drug unassisted, having been provided with comprehensive advice on this at the time of their recruitment. However, a back-up service will also be in place: if they are in any doubt, parents or guardians will be instructed to contact their local study site, or if this is not possible, to call a PREDNOS 2 study telephone number. This will be manned by the chief investigator or his nominated deputy during periods of annual leave. Through this service, parents or guardians will be able to seek advice regarding whether their child meets the URTI criteria, the dose of study drug required, and any other issues or concerns they may have relating to the study. A log of these telephone calls will be maintained, and the chief investigator will report their content to the local principal investigator by email.

To ensure patient safety, the information provided to parents and guardians will also contain information about the signs of a more serious infection (non-blanching rash, leg pain, cool extremities, rapid breathing, blue lips, fitting, unconsciousness or any other major concern). If any of these features are present, parents and guardians will be instructed not to start the study drug and to seek urgent medical attention for their child from their general practitioner or local accident and emergency department. Parents or guardians will be asked to contact their local study site within 24 hours of commencing the study drug to inform the principal investigator or nominated deputy and to allow them the opportunity to discuss any of their child’s symptoms that may be of concern to them.

### Further commencement of study drug

The above intervention will be repeated every time the child develops an URTI over the 12-month follow-up period. The only exception to this is if the child is receiving daily prednisolone therapy, e.g. in the early stages of treatment for a previous relapse – in this instance the study drug will not be commenced.

### Dosing of study drug

The precise dose of the study drug at time of the development of URTI will depend upon the subject’s current treatment regimen, in particular whether they are receiving long-term maintenance prednisolone therapy and if so, at what dose.

#### Subjects not receiving long-term maintenance prednisolone

Those randomised to the active treatment arm will receive prednisolone 15 mg/m^2^ daily (maximum dose 40 mg) for a total of 6 days. The dose will be rounded up or down to the nearest 5 mg and given as a single morning dose. Subjects randomised to the control arm will receive an identical number of placebo tablets.

#### Subjects receiving a long-term maintenance prednisolone dose of ≤15 mg/m^2^ on alternate days

Those randomised to the active treatment arm will receive prednisolone 15 mg/m^2^ daily (maximum dose 40 mg) for a total of 6 days. The dose will be rounded up or down to the nearest 5 mg and given as a single morning dose. Subjects randomised to the control arm will receive an identical number of placebo tablets.

These subjects receiving long-term maintenance prednisolone therapy will receive a different number of tablets of the study drug on regular treatment days (i.e. those days when they usually take prednisolone) from non-treatment days. For example, a child of 1.0 m^2^ receiving a long-term maintenance dose of 5 mg of prednisolone on alternate days will take an additional 2 × 5 mg prednisolone tablets (or matching placebo) on treatment days and 3 × 5 mg prednisolone tablets (or matching placebo) on non-treatment days. This particular subject would therefore either receive prednisolone 15 mg (15 mg/m^2^) daily for 6 days if in the active treatment arm or continue unchanged on prednisolone 5 mg on alternate days if in the control arm.

#### Subjects receiving a prednisolone dose of >15 mg/m^2^

A small number of subjects will be receiving a prednisolone dose of >15 mg/m^2^ at the time of URTI development. They will have already relapsed during the 12-month study period and had their prednisolone dose increased accordingly as the inclusion/exclusion criteria exclude those on a dose of prednisolone >15 mg/m^2^ at study entry. These subjects will convert to the same dose on a daily basis for a total of 6 days. The dose will be rounded up or down to the nearest 5 mg and given as a single morning dose (maximum dose 60 mg). Subjects randomised to the control arm will receive an identical number of placebo tablets. For example, a child of 1.0 m^2^ receiving a long-term maintenance dose of 20 mg of prednisolone on alternate days will take their regular dose on treatment days and 4 × 5 mg prednisolone tablets (or matching placebo) on non-treatment days. This particular subject would therefore either receive 20 mg (20 mg/m^2^) daily for 6 days if in the active treatment arm or continue unchanged on 20 mg on alternate days if in the control arm.

The study drug dose to be used on development of an URTI may change during the study due to alterations in the subject’s long-term maintenance prednisolone dose and normal childhood growth. The precise regimen to be administered will be discussed with parents and guardians along with a written treatment plan at the time of recruitment and (in light of the above) will be re-discussed at each study visit. Information about the precise number of study drug tablets to administer will be provided to the family in written form, using a standard form, which will be completed by the local principal investigator.

The study drug will always be given as a single dose in the morning. If the subject is receiving an additional immunomodulatory therapy, e.g. ciclosporin, levamisole, etc., this will continue unchanged throughout the 6-day course of the study drug. Other drug treatment will also continue unchanged. At the end of the 6-day course of the study drug, the subject will continue on their previous dose of long-term maintenance prednisolone therapy (or no prednisolone treatment if they were not previously receiving this). Other drug treatment will continue unchanged.

When URTI occurs whilst children are receiving daily prednisolone, e.g. in the early stages of treatment for a disease relapse, the study drug will not be commenced. Subjects can continue to participate in the study and the subsequent URTIs will be treated with the study drug provided that they are receiving alternate-day prednisolone at this point.

### Information recorded by parents and guardians

Parents or guardians will perform dipstick testing for proteinuria (using an Albustix, Siemens Healthcare Diagnostics Ltd, Frimley, UK) of the subject’s first morning urine on a daily basis in accordance with routine clinical care. They will be provided with a subject-held record book to enter the results (to allow the early detection of nephrotic syndrome relapses) and the medication administered on a daily basis. This will be maintained for the 12 months of the study. Parents and guardians will also use this diary to record any intercurrent illness and consultations with healthcare professionals (general practitioner, nurse, hospital accident and emergency department, etc.), development of URTI, and commencement of study medicines, along with details of medicines prescribed or purchased over the counter.

### Diagnosis and treatment of relapse

Relapse is defined as Albustix +++ (3+) or more for three consecutive days or the presence of generalised oedema and Albustix 3+ on urine testing. An URTI-related relapse is defined as a relapse occurring within 14 days of the onset of an URTI. Where disease relapse occurs, parents or guardians will contact their study centre in accordance with routine clinical care and treatment for disease relapse will be commenced. A relapse will be treated in accordance with the International Study of Kidney Disease in Children relapse regimen: prednisolone will be commenced at a dose of 60 mg/m^2^ daily (maximum dose 80 mg) until the urine tests are negative or trace for three consecutive days, then reduced to 40 mg/m^2^ (maximum dose 60 mg) on alternate days for 4 weeks (14 doses). A subsequent tapering dose can be used at the individual physician’s discretion.

When relapse therapy is commenced, long-term maintenance prednisolone therapy (e.g. 10 mg on alternate days) is discontinued. Where relapse occurs whilst the child is receiving the 6-day course of the study drug, this will also be discontinued. Once the relapse regimen has been completed, long-term maintenance prednisolone therapy may be recommenced at any dose at the principal investigator’s discretion. Background immunomodulatory therapy other than prednisolone, e.g. ciclosporin, mycophenolate mofetil, levamisole, etc., will continue unchanged throughout the relapse treatment period.

### Escalation of background immunomodulatory therapy

Subjects will only undergo intensification of background immunomodulatory therapy (i.e. the addition of, or change to, a new immunomodulatory agent e.g. ciclosporin, mycophenolate mofetil, levamisole, etc.) when there are two or more relapses of their nephrotic syndrome (URTI-related or unrelated) in any 6-month period or where there are unacceptable adverse effects of prednisolone or other therapy. These subjects will remain under follow-up: intensification of immunomodulatory therapy is an important secondary end point for this study. Similarly, immunomodulatory therapy will only be discontinued when the subject has remained relapse-free for at least 6 months or where there are unacceptable adverse effects of therapy.

### Trial schema and study visit schedule

All subjects will undergo comprehensive assessment at the randomisation visit and will then be followed up for 12 months from randomisation, with visits once every 3 months at 3, 6, 9 and 12 months for trial follow-up assessments. The information captured at the randomisation and subsequent visits are shown in Table 1. At each visit, there will be a full clinical review of the subject, and a number of different questionnaires will be administered. The clinical review will include assessment of whether the subject has had any URTI or non-URTI related relapses since the last visit, and a review of the current treatment regimen. To evaluate changes in subject behaviour associated with the different prednisolone regimens, the Achenbach Child Behaviour Checklist will be used. This is a standardised measure made up of 120 items measuring internalising (withdrawn, somatic complaints, anxiety/depression, thought problems) and externalising (social problems, attention problems, delinquent and aggressive) behaviour problems. A total Behavioural Problem score is calculated from these problem scales and forms the basis of comparison with age- and gender-matched normative data. Information relating to quality of life will also be collected using the Child Health Utility 9D (CHU-9D), EQ-5D (dependent upon subject age) and PedsQL questionnaires. The CHU-9D is a newly developed utility measure designed for children aged 5 to 11 years. The EQ-5D is a validated utility measure routinely used in adult and adolescent populations. Both the CHU-9D and the EQ-5D will be used to generate data for the health economic analyses. The PedsQL questionnaire is a well-validated approach to measuring health-related quality of life in healthy children and adolescents and those with acute and chronic health conditions.

**Table 1:** Study visit schedule

**Visit**	**1**	**2**	**3**	**4**	**5**
Month	0	3	6	9	12
Visit window		+/-2 weeks	+/-2 weeks	+/-2 weeks	+/-2 weeks
Inclusion/exclusion criteria	x				
Informed consent	x				
Randomisation (post-recruitment)	x				
Allocation of study number	x				
Documentation of URTI		x	x	x	x
Documentation of commencement of study drug		x	x	x	x
Documentation of recent relapse	x	x	x	x	x
Recent medical and drug history	x	x	x	x	x
Adverse event documentation		x	x	x	x
Compliance check (tablet count using counting triangle)		x	x	x	x
Physical exam	x	x	x	x	x
Assessment of steroid toxicity	x	x	x	x	x
Height and weight	x	x	x	x	x
Blood pressure	x	x	x	x	
Calculation of study drug dose to be administered in event of URTI and explanation and provision of documentation of this to parents and guardians – includes review of height, weight and body surface area to confirm correct dose	x	x	x	x	x
If three or more courses of study drug have been administered since previous visit, confirm parental understanding of definition of URTI		x	x	x	x
Blood sample for DNA/RNA^a^	x	x	x	x	x
Achenbach Child Behaviour Checklist	x	x	x	x	x
PedsQL questionnaire	x	x	x	x	x
CHU-9D and EQ-5D questionnaires	x	x	x	x	x
Study drug returned to central pharmacy for accountability					x

^a^Blood sample to be collected on one single occasion only.

URTI, upper respiratory tract infection.

Adverse effects due to prednisolone will be assessed by studying growth (height, weight and body mass index), Cushingoid features, hypertrichosis, striae, appetite (all Likert scale), behaviour (Achenbach Child Behaviour Checklist), blood pressure and urine dipstick analysis for glycosuria. The development of significant bacterial, viral or fungal infections and the use of post-varicella exposure prophylaxis (zoster immune globulin or antiviral therapy) will also be recorded. Information will also be collected regarding all episodes of consultation with general practitioners or hospital medical teams, including data on treatments prescribed or purchased. This will be incorporated into the costs to be measured as part of the health economic analysis (see below).

A single 10 ml blood sample will be collected on one occasion for those who provide additional consent for this. DNA and RNA will be extracted for genetic sub-studies. A genome-wide association study will be used to identify possible genetic loci associated with SSNS. Additionally, a rapid screen using direct Sanger sequencing will identify whether mutations in any of the known genes cloned to date in connection with childhood nephrotic syndrome are present. If pathogenic variants are absent, samples will then be subjected to whole exome analysis.

### Subject retention

This visit schedule is in keeping with the frequency of routine follow-up of children with relapsing SSNS. As such, we anticipate high rates of subject retention. In the PREDNOS study (ISRCTN16645249), which has to date recruited almost 200 newly presenting cases of SSNS, our retention rate is currently close to 90%. The large majority of PREDNOS investigators are also participating in the PREDNOS 2 study.

### Sample size and power calculation

A total of 300 subjects will be recruited into the study (150 in each study arm). The primary analysis will be based on a comparison of the number of URTI-related relapses of nephrotic syndrome in the two study arms following the first URTI during the 12-month follow-up period. In children with frequently relapsing SSNS, the development of an URTI results in relapse in around 50% of instances [7]. In the Abeyagunawardena study, overall 40 URTIs treated with placebo were followed by 19 (48%) relapses, compared with 7 (18%) relapses in the prednisolone-treated group. This corresponds to an absolute difference of 30% (a 62.5% proportional reduction). In the first treatment period, there were 10 (45%) relapses for 22 placebo-treated children compared with 4 (22%) relapses for 18 prednisolone-treated children (23% absolute difference, 51% proportional reduction) [9]. This is a large treatment effect based on a small study of children on long-term maintenance alternate-day prednisolone therapy in a developing country. Therefore, to detect a more conservative difference of 17.5% (i.e. 35% proportional reduction) in URTI-related relapse rate (i.e. from 50% to 32.5%), with 80% power, 2-sided test and alpha of 0.05, requires 250 subjects in total (comparison of two proportions [11]). If allowance is made for between 10% and 20% dropout (e.g. subject withdrawal, lost to follow-up or subject not having an URTI during the 12-month follow-up period), then this will require recruitment of between 280 and 320 subjects. We therefore propose to recruit 300 subjects, 150 to each arm. However, if the treatment effect is more in line with the 50% reduction observed in the first treatment period of the Abeyagunawardena study, then this study has sufficient power (>95%) to detect this difference. To detect a 50% proportional reduction (i.e. 50% to 25%) with 90% power and alpha of 0.05, requires 160 subjects, increasing to 200 with allowance for 20% dropout.

### Outcome measures

#### Primary study end point

The primary study end point is URTI-related relapse of nephrotic syndrome following the first URTI during the 12-month follow-up period. An URTI-related relapse is defined as a relapse occurring within 14 days of the onset of URTI.

#### Secondary end points

The secondary end points are:

• Rate of URTI-related relapse of nephrotic syndrome (relapses per year)

• Rate of relapse (URTI-related and non URTI-related) of nephrotic syndrome (relapses per year)

• Cumulative dose of prednisolone (mg/kg and mg/m^2^) received over the 12-month study period

• Incidence of serious adverse events

• Incidence of adverse effects due to prednisolone based on assessment of behaviour using the Achenbach Child Behaviour Checklist

• Incidence of escalation of background immunomodulatory therapy (e.g. addition of ciclosporin, tacrolimus, cyclophosphamide, etc.)

• Incidence of reduction of background immunomodulatory therapy (i.e. cessation of long-term maintenance prednisolone therapy)

• Quality of life using CHU-9D, EQ-5D and PedsQL

• Cost per relapse of nephrotic syndrome

• Cost per quality adjusted life year (QALY) gained

### Statistical analysis

The statistical analysis plan describes the planned analyses for PREDNOS 2 in full detail. A summary of the main analysis methods are described here. The primary end point is URTI-related relapse of nephrotic syndrome following the first URTI during the 12-month follow-up period. The proportion of subjects that experience an URTI-related relapse will be calculated for the two study treatment arms, and compared using a chi-squared test. Treatment effects will be expressed as relative risks with 95% confidence intervals. This was chosen as the primary end point as it is hypothesised that giving daily prednisolone during an URTI will reduce the subsequent development of disease relapse. If this hypothesis is correct, then those subjects randomised to the placebo may experience more disease relapses than those subjects randomised to the active drug. This would mean that subjects in the placebo arm are more likely to undergo intensification of immunomodulatory therapy. Previous studies excluded these subjects from the study and data analysis, as escalating therapy may mean that the overall likelihood of these children relapsing is reduced, which could introduce bias into the analysis. In PREDNOS 2, a pragmatic decision has therefore been taken to continue to follow up these subjects as it is not known whether these assumptions hold true, and by excluding these subjects it also introduces bias through the differential dropout between the two treatment arms. Therefore, all subjects will be followed up for 12 months to also assess the relapse rates between the two treatment arms over a 12-month period as secondary end points, however,due to these potential biases, the primary end point will focus on the relapse rate following the first URTI. The secondary end points relating to relapses per year will assess incidence (relapse) density rates for the two treatment arms and rate differences will be calculated for both URTI- and non-URTI-related relapse of nephrotic syndrome over the 12-month follow-up period. An appropriate regression analysis (e.g. Poisson) will be used to compare relapse rates in the two treatment groups should there be an imbalance in any important covariates (e.g. URTI rates across the two study arms).

The secondary end points include both continuous and categorical data items. The dichotomous data (e.g. incidence of escalation of background immunosuppressive therapy) will be analysed as per the primary outcome. Continuous data (e.g. prednisolone dose and quality of life scores) will be expressed as means and standard deviations, with differences in means and 95% confidence intervals. Longitudinal plots of the data over time will be constructed to give a visual presentation of the data. The data will be analysed using mixed effect repeated measures models with the interventions specified as independent variables. Baseline data will be included in the model as a covariate. Analyses will be of all randomised subjects using intention to treat, except for those who do not have an URTI following randomisation (estimated to be <10%). Exclusion of these subjects will result in no bias as these dropouts are not related to the treatment and should by chance be equally distributed across the two treatment arms (this will be monitored throughout the trial). The only subgroup analyses planned are an analysis of the background treatment regimen that the subjects are on at randomisation (no long-term treatment, on long-term maintenance prednisolone therapy, on other immunosuppressant therapy with long-term maintenance prednisolone therapy or on other immunosuppressant therapy without long-term maintenance prednisolone), an analysis comparing those subjects on long-term maintenance prednisolone versus those not on long-term maintenance prednisolone and an analysis comparing those subjects on other immunomodulatory therapy versus those not on other immunomodulatory therapy. There is no evidence to suggest that any difference in treatment effect will occur and the study is not powered to detect differences in treatment effect across these subgroups; these analyses will be considered as hypothesis generating.

### Health economic analysis

The economic evaluation will take the form of a cost-effectiveness analysis based on a primary clinical outcome of cost per episode of URTI-related relapse of proteinuria. Utility-based outcomes will also be incorporated into the model allowing a secondary outcome to be cost per QALY gained (based on CHU-9D and EQ-5D values). Four different groups of subjects will be considered in the model: (i) subjects not on long-term immunomodulatory therapy, (ii) subjects receiving long-term maintenance prednisolone therapy, (iii) subjects receiving long-term maintenance prednisolone therapy in conjunction with other immunomodulatory therapies and (iv) subjects receiving long-term immunomodulatory therapies without long-term maintenance prednisolone therapy. For each of these four groups, the model will make a direct comparison between two strategies implemented each time a subject develops an URTI: the strategy of administering a course of daily prednisolone for 6 days and a strategy of no change to ongoing therapy (with the use of placebo tablets to maintain blinding). The base case economic evaluation will adopt the National Health Service (NHS) perspective. NHS costs will include:

• Treatment costs: medicines, management, adverse-effects and treatment complications

• Consultation and follow-up costs: routine tests such as blood tests and urinalysis, number of outpatient visits, inpatient visits and general practitioner visits

• Longer-term treatment costs (care for long-term adverse effects)

Resource-use data such as visits to the subject’s general practitioner, hospital (as an outpatient or inpatient) and medicines supplied will be collected using subject-held record books provided for them. This information will be collected at the time of study visits. Due to the economic burden associated with relapse of URTI-related proteinuria that is placed on patients and their families, we will also conduct an economic evaluation that includes private costs such as over-the-counter medicines and parents’ or guardians’ time off work. Resource-use data related to these costs will also be collected using patient-held record books. Unit cost data for both NHS and private costs will be derived from nationally representative sources such as the British National Formulary, the National Schedule for Reference Costs and the Unit Costs of Health and Social Care.

As there are no utility measures validated for use across the complete age range of the study population (1 to 18 years), the economic analysis will use a combination of instruments including the CHU-9D, the EQ-5D and the PedsQL. This combined approach will allow for a methodological exploration into the use of utility measures in this age group whilst ensuring that appropriate information is collected to facilitate the construction of QALYs.

To test the robustness of the conclusions to assumptions made in the modelling, and to sampling variation in the data used in the construction of the model, a full deterministic and probabilistic sensitivity analysis will be carried out and results will be reported in terms of incremental cost-effectiveness ratios and cost-effectiveness acceptability curves. Costs and benefits will be discounted at the standard rate (3.5%).

### Ethical considerations

Children are considered vulnerable trial subjects; however, a trial involving children with nephrotic syndrome is ethically justified as the condition is specific to children and the evidence base for treatment used in clinical practice is inadequate. It is not possible to perform the study in adult subjects and extrapolate the results to children. The active treatment arm will receive a 6-day course of daily prednisolone at the time of each URTI, which they develop over the 12-month study period. Our hypothesis is that this will reduce the risk of subsequent relapse and reduce the total amount of prednisolone that these subjects are exposed to. There is a risk that the hypothesis is incorrect and that these subjects will therefore receive unnecessary additional prednisolone treatment with its attendant adverse effects. The subjects participating in the study will already be receiving significant annual total doses of prednisolone as a result of their relapsing disease and the additional prednisolone administered through their participation in PREDNOS 2 will represent only a relatively small increase in total annual dose. We will carefully monitor adverse effects of prednisolone in this study, including the effect on child behaviour.

### Reporting of adverse events

Pharmacovigilance reporting will comply with the Medicines for Human Use (Clinical Trials) Regulations 2004 and Amended Regulations 2006. Annual development safety update reports will be submitted to the main research ethics committee and the Medicines and Healthcare Products Regulatory Authority. Within the PREDNOS 2 trial, prednisolone and the matching placebo are both defined as investigational medicinal products. Serious adverse events will be reported for the duration of the 12-month study and for 3 months following treatment with the study drug.

### Monitoring

#### Trial steering committee

The independent members of the trial steering committee are Dr Megan Thomas (independent expert and chair), Dr Nigel Coad (independent expert), Mrs Sandra Cope (independent consumer representative), Dr Andrew Duncan (independent expert), Dr Kate Hillman (independent expert), Dr Zala Ibrahim (independent expert) and Dr Ly-Mee Yu (independent expert statistician). Additional non-independent members of the trial steering committee are Miss Natalie Ives (BCTU statistician) and Professor Nicholas Webb (chief investigator).

Additional trial steering committee attendees are Miss Emma Barsoum (BCTU study manager), Mrs Elizabeth Brettell (BCTU renal trials manager), Dr Martin Christian (principal investigator), Professor Peter Clayton (external expert), Mrs Wendy Cook (consumer representative), Dr Richard Coward (investigator), Dr Carol Cummins (external expert), Dr Emma Frew (health economist), Mr Terry Hughes (BCTU renal data manager), Miss Charmaine Meek (BCTU renal data manager), Dr Richard Trompeter (external expert) and Miss Rebecca Wooley (statistician).

#### Independent data monitoring committee

The members of the independent data monitoring committee are Professor Philip Kalra (chair), Professor Saul Faust and Dr Andrea Marshall.

### Regulatory aspects

PREDNOS 2 is sponsored by the Central Manchester University Hospitals NHS Foundation Trust and the University of Birmingham (RG_12-188). The Medicines and Healthcare Products Regulatory Authority clinical trial authorisation reference is 21761/0281/001-0001. The EudraCT number is 2012-003476-39. The active study drug and placebo are manufactured in accordance with current Good Manufacturing Process regulations. The study will be conducted in compliance with the Research Governance Framework for Health and Social Care, the Medicine for Human Use (Clinical Trials) Regulation 2004 and Good Clinical Practice.

### Ethical committee approval

Ethical approval for PREDNOS 2 was granted by the National Research Ethics Service Committee North West – Greater Manchester Central (12/NW/0766) on 4 December 2012.

## Discussion

### Potential impact

There is substantial disease- and treatment-related morbidity associated with disease relapses in SSNS. PREDNOS 2 will be the largest ever study to investigate whether the administration of a 6-day course of daily prednisolone therapy given at the time of onset of URTI prevents the subsequent development of a disease relapse. Furthermore, the study will include children with SSNS receiving a wide range of long-term maintenance immunomodulatory therapies, in contrast to previous studies, which focused upon children receiving long-term maintenance prednisolone and levamisole only. If it is possible to reduce the disease relapse rate effectively and safely using the intervention being tested in PREDNOS 2, this will reduce relapse-associated morbidity and the cost associated with drug treatment, possible hospital admission and parental absence from employment. PREDNOS 2 will be the first study in patients with relapsing SSNS that will incorporate a formal health economic analysis and investigate quality of life.

The proposed intervention (6 days of daily prednisolone) is simple to administer and would be easy to introduce into routine clinical practice were the results of this study to recommend its use. The parents and guardians of children with SSNS are used to introducing changes to prednisolone treatment based upon the results of urinalysis performed in the home, and maintain supplies of the drug in the home for use at such times.

The study also proposes to use additional analyses to examine whether genetic background and/or deleterious gene variants result in SSNS, beyond genes already known to be associated with certain types of childhood nephrotic syndrome. It is anticipated that these may well provide valuable information about disease pathogenesis, as well as potentially predict disease progression, either through contributing to our existing knowledge on podocyte dysfunction or by indicating which patients may respond better to medication.

### Dissemination

The study results will be published in accordance with the CONSORT statement and SPIRIT guidelines [12,13]. Our findings will be submitted to major international paediatric nephrology and general paediatric meetings, and submitted for publication in a high impact factor journal, with open access. The results will also be communicated in plain English to study participants and their families.

## Trial status

As of 2 April 2014 a total of 80 children have been recruited into the study.

## Abbreviations

BCTU: Birmingham Clinical Trials Unit; CHU-9D: Child Health Utility 9D; NHS: National Health Service; NIHR: National Institute for Health Research; QALY: quality adjusted life year; REC: research ethics committee; SSNS: steroid-sensitive nephrotic syndrome; URTI: upper respiratory tract infection.

## Competing interests

The authors declare that they have no competing interests.

## Authors’ contributions

NJAW conceived the study, led its design and co-ordination and drafted the manuscript. NJI participated in the design of the study, led the planned statistical analysis and contributed to the writing of the manuscript. EF participated in the design of the study, led the health economic evaluation and contributed to the writing of the manuscript. DB, AK and MAS participated in the design of the study, led the genetic sub-studies and contributed to the writing of the manuscript. EAB, DVM, MC, ASH, HM, SH, ERF, RDG, JB, CJ, KM and WC participated in the design of the study and contributed to the writing of the manuscript. All authors read and approved the final manuscript.
